# Functional and electron-microscopic changes after differential traction injury in the sciatic nerve of a rat

**DOI:** 10.1186/s40902-021-00297-4

**Published:** 2021-05-01

**Authors:** Soo-Hwan Byun, Kang-Min Ahn

**Affiliations:** 1 Department of Oral & Maxillofacial Surgery, Department of Dentistry, Hallym University Sacred Heart Hospital, Anyang-si, Republic of Korea; 2 Department of Oral and Maxillofacial Surgery, College of Medicine, University of Ulsan, Asan Medical Center, 88 Olympic-ro, 43-gil, Songpa-gu, Seoul, 05505 Republic of Korea

**Keywords:** Sciatic nerve, Tension, Injury, Stretch, Peripheral nerve, Axon, Gait analysis

## Abstract

**Background:**

During maxillofacial trauma or oral cancer surgery, peripheral nerve might be damaged by traction injury. The purpose of this study was to evaluate functional and histomorphometric changes after traction nerve injury in the sciatic nerve of a rat model.

**Methods:**

A total of 24 Sprague-Dawley rats were equally divided into three groups: unstretched (sham/control, group A), stretched with 0.7N (group B) and 1.5N (group C). Traction injury was performed for 10 min in B and C groups. Functional recovery of the sciatic nerve was evaluated by walking track analysis, toe spread test, and pinprick test 2 weeks after injury. The weight of gastrocnemius muscles of both sides was measured to evaluate weight ratio (ipsilateral/contralateral). Total number of axons, axon fiber size, myelin thickness, G-ratio, axon number/mm^2^, diameter of fiber, changes of longitudinal width, and formation of the edema and hematoma were evaluated by transmission electron microscopy.

**Results:**

The sciatic function indexes were −11.48±4.0, −15.11±14.84, and −49.12±35.42 for groups A, B, and C, respectively. Pinprick test showed 3.0, 2.86±0.38, and 1.38±0.52 for A, B, and group C. Muscle weight ratios were 0.98±0.13 for group A, 0.70±0.10 for group B, and 0.54±0.05 for group C. There were significant differences in toe spread test, pinprick test, and muscle weight ratio between control group and experimental group (*p*<0.001). In the experimental group, fiber number, fiber size, G-ratio, fiber number/mm^2^, myelin thickness, diameter of fiber, and longitudinal width were decreased with statistical significance.

**Conclusion:**

The present study demonstrated that the nerve traction injury in the rat sciatic nerve damaged the motor and sensory function and axonal integrity. The amount of functional nerve damage was proportional to the amount of traction power and dependent on the initial tensile strengths (0.7N and 1.5N).

## Bacground

Traction nerve damage is one of the most common complications in maxillofacial surgeries such as oral cancer operation, parotidectomy, genioplasty, maxillofacial fracture reduction, and free flap elevation [1–5]. These retraction injuries induce nerve elongation (15%), ischemia, histological change (4–50%), and mechanical failure (30–73%) of the peripheral nerves [6–9]. Peripheral nerve discontinuation due to traction injury may happen between 30 and 73% elongation [9]. Therefore, it is crucial to evaluate the severity of nerve damage during surgical procedures to predict functional recovery of the nerve.

If the clinician diagnoses the nerve damage, various treatments such as medication, physical therapy, or electro-stimulator therapy could be applied [10]. However, the prognosis is largely dependent on the initial strength of the traction and anatomical damage. Therefore, it is important to predict the severity of nerve damage in accordance with strength of the injury. There were studies about nerve damage and prognosis after traction injury less than 1.0 N. The threshold identified for rat sciatic nerve was between 0.41±0.02N and 0.50±0.06N [11]. However, there was no study about nerve damage after traction injury more than 1.0N which might cause mechanical failure or nerve discontinuation. The purpose of this study was to evaluate the functional recovery and nerve histomorphometric changes following low (0.7 N) and high (1.5 N) grade nerve traction injuries.

## Materials and methods

### Surgical procedure

**Fig. 1:**
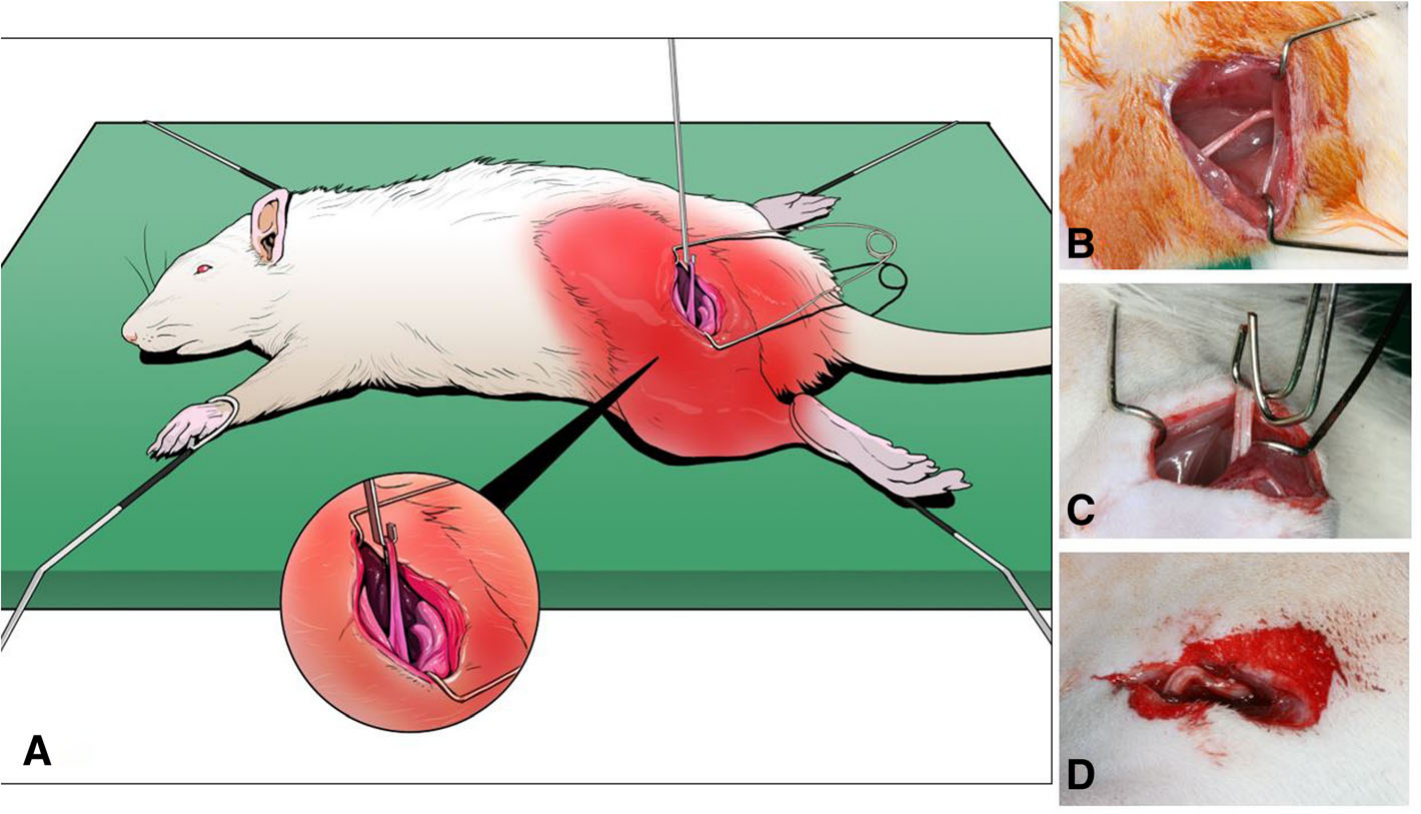
Surgical procedure for traction injury of the sciatic nerve of a rat. (**a** schematic drawing of the experiment, **b** exposure of the sciatic nerve, **c** traction injury with hook wire, **d** Elongated nerve after traction injury)

Twenty-four male Sprague-Dawley rats weighing 200 to 300 g were included in the study. Animals were divided into 3 groups (*n*=8 per group). In group A, the sciatic nerve was just exposed for 10 min while in the other two groups the sciatic nerve was stretched by 0.7 N and 1.5 N (groups B and C, respectively) for 10 min using a spring-balance device (Fig. 1). This animal experiment was approved by the ethics committee on the experimental use of laboratory animals of the Asan Medical Center Animal Research Committee.

The detailed surgical procedures were as follows. Animals were anesthetized with sodium pentobarbital (Thiopental sodium™ 300mg/kg ip, Choong Wae Pharm, Korea). After routine povidone iodine (Betadine™, Choong Wae Pharm, Korea) preparation of the operative field on the left thighs, the rats’ limbs were fixed at the experimental plate. The left sciatic nerves were exposed through a 1.5-cm straight skin incision at the posterior surface of the upper thigh. After intermuscular septal dissection, a 10-mm segment of the sciatic nerve was dissected and marked with indelible ink to measure the nerve length change. Following that, the tension was removed and the nerve carefully detached from the spring-balance and returned to its original bed. The occurrence of nerve rupture or discontinuity was examined with magnifying loupe in groups B and C, and elongation of the nerve was recorded. The flap was sutured with 4-0 Vicryl™ (Ethicon, UK) by layered suturing technique. Antibiotic (Amoxicillin™ 150 mg/kg, Il Sung Pharm, Korea) and analgesic (Ketoprofen™ 2–5 mg/kg, Dong Kook Pharm, Korea) were injected intramuscularly after the procedure every 12 to 24 h for 1 week.

### Functional evaluation

**Fig. 2:**
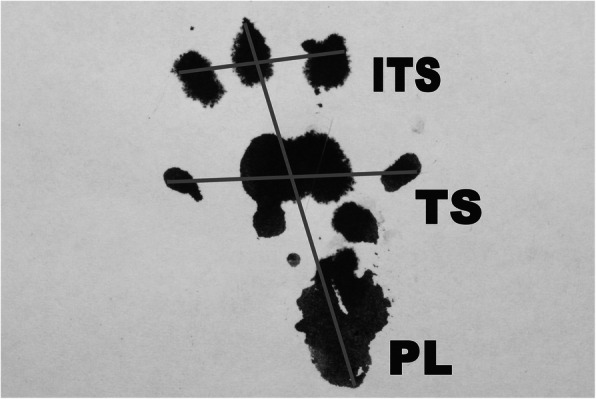
Normal footprint after walking track analysis. (ITS intermediary toe spread, TS toe spread, PL print length)

Function of the sciatic nerve was evaluated 2 weeks after the injury using the pinprick test, toe-spreading test, and walking track analysis as explained below. Moreover, the gastrocnemius muscle weights were measured on both sides to evaluate the effect of denervation on muscle volume. Pin prick test was used to assess the recovery of sensory function. In the upside-down controlling position, knees, ankles, and feet were pinned by a 21-gauge needle. The response was graded on a 0 to 3 point scale: 0, no reaction to the stimulant; 1, sensation above the knee level; 2, sensation between the knee and ankle; and 3, sensation distal to the ankle [12]. The toe-spreading was used to assess motor function recovery. The test evaluates the voluntary reaction in response to raising the animal by the tail. The test score ranges from 0 to 3 (no reaction, 0; any sign of movement, 1; abduction of the tow, 2; extension of the toe, 3) [12]. Functional analysis was also performed using the walking track test. Footprints of the rats were recorded while they were walking on a corridor made out of white paper. The following parameters were obtained from the foot prints: distance from the heel to the top of the third toe (print length; PL), distance between the first and the fifth toe (toe-spreading; TS), and distance from the second to the fourth toe (intermediary toe-spreading; IT) (Fig. 2). These measures were taken both from the experimental sides (EPL, ETS, EIT, respectively) and from the non-operated side (NPL, NTS, and NIT, respectively). Following these measurements, sciatic function index (SFI) was calculated using the following formula [13–15]. A SFI score of 0 is considered normal function, while a SFI score of −100 indicated total impairment caused by a complete transection of the sciatic nerve.

$$ \mathrm{SFI}=-38.3\left(\mathrm{EPL}-\mathrm{NPL}\right)/\mathrm{NPL}+109.5\left(\mathrm{ETS}-\mathrm{NTS}\right)/\mathrm{NTS}+13.3\left(\mathrm{EIT}-\mathrm{NIT}\right)/\mathrm{NIT}-8.8. $$

### Muscle weight ratio evaluation

The gastrocnemius muscle weight was measured to assess the atrophy resulted by denervation of the muscle 2 weeks post-surgery. The gastrocnemius muscles were excised from both sides, and muscle weights were measured immediately. Wet weight of the experimental muscles was compared to the contralateral side, and muscle weight index was calculated [12].

### Histomorphometric evaluation

The animals were euthanized with CO_2_ gas 2 weeks after tension injury, and all of the sciatic nerves were harvested. The samples from two rats in each group were used to make longitudinal sections and stained with H&E. Two to four longitudinal sections were obtained from each sample, which were visualized using a slide scanner (Photosmart S20™, Hewlett Packard). The changes of the width, separation between fibers, and discontinuity of the nerves were analyzed. Samples from six rats in each group were examined under transmission electron microscope (TEM) to measure total number of axons, fiber density, relative sheath thickness (G-ratio), and myelin thickness. After sample preparation, transverse semi-thin (5-μm-thick) sections were cut using a microtome and stained with toluidine blue. Representative areas were selected under the light microscope, and six randomized TEM photos were obtained from each sample using electron microscope (×2500, JEOL 1200 EX-II™, Japan). The density of the nerve fiber was estimated by using a randomized counting frame. The total axon number and axonal density (axon number/mm^2^) were counted in the endoneurial areas surrounded by the inner border of perineurium and the counting frame. The axons on the frame border or the perineurium were not counted. The myelin thickness (μm) was determined by deducting the area of the axon from the area of the whole fiber profile containing the axon. The thickness of the myelin sheath at the most compact and the least compact region was measured. In the myelinated axons, four different areas were selected, and the mean values between them were calculated. The G-ratio of the myelinated fiber was obtained by dividing the diameter of axon by the diameter of the fiber. The G-ratio ranges from 0 to 1, 1 being unmyelinated axon.

### Statistical analysis

The Kruskal-Wallis test was used to compare mean values of the three groups, and *p*<0.05 was considered statistically significant. The Mann-Whitney test was used to compare the mean values of the groups two by two, and *p*<0.0167 (0.05/3: Bonferroni correction) was considered statistically significant.

## Results

### Function and muscle evaluation

**Fig. 3:**
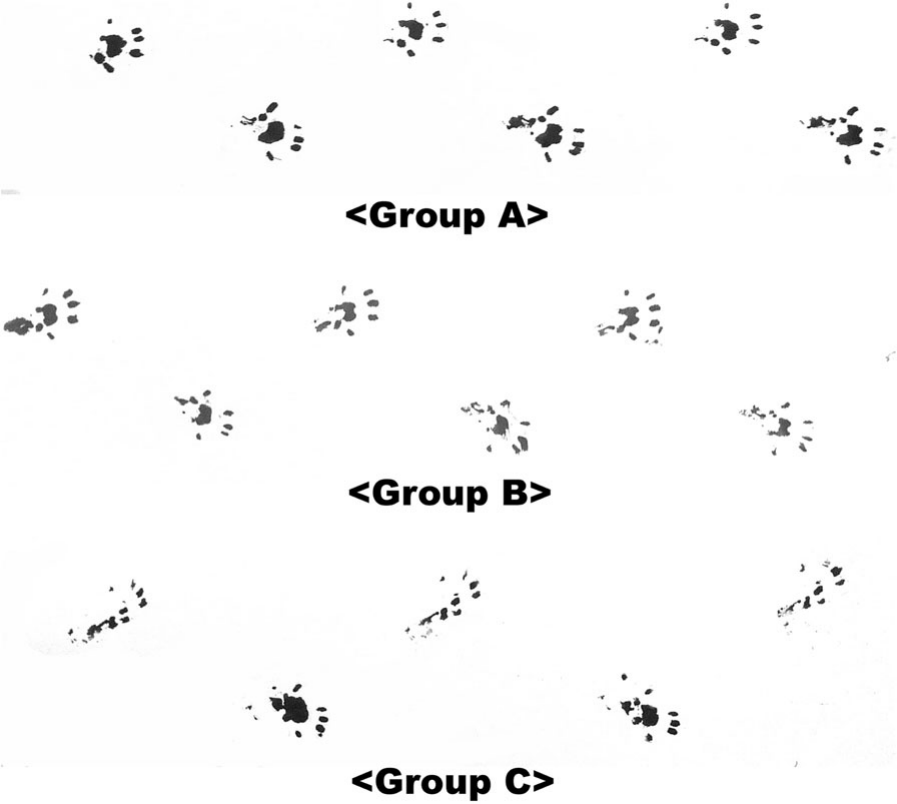
Footprints of sham operation (group A), 0.7 N traction injury (group B), and 1.5 N traction injury (group C)

**Fig. 4:**
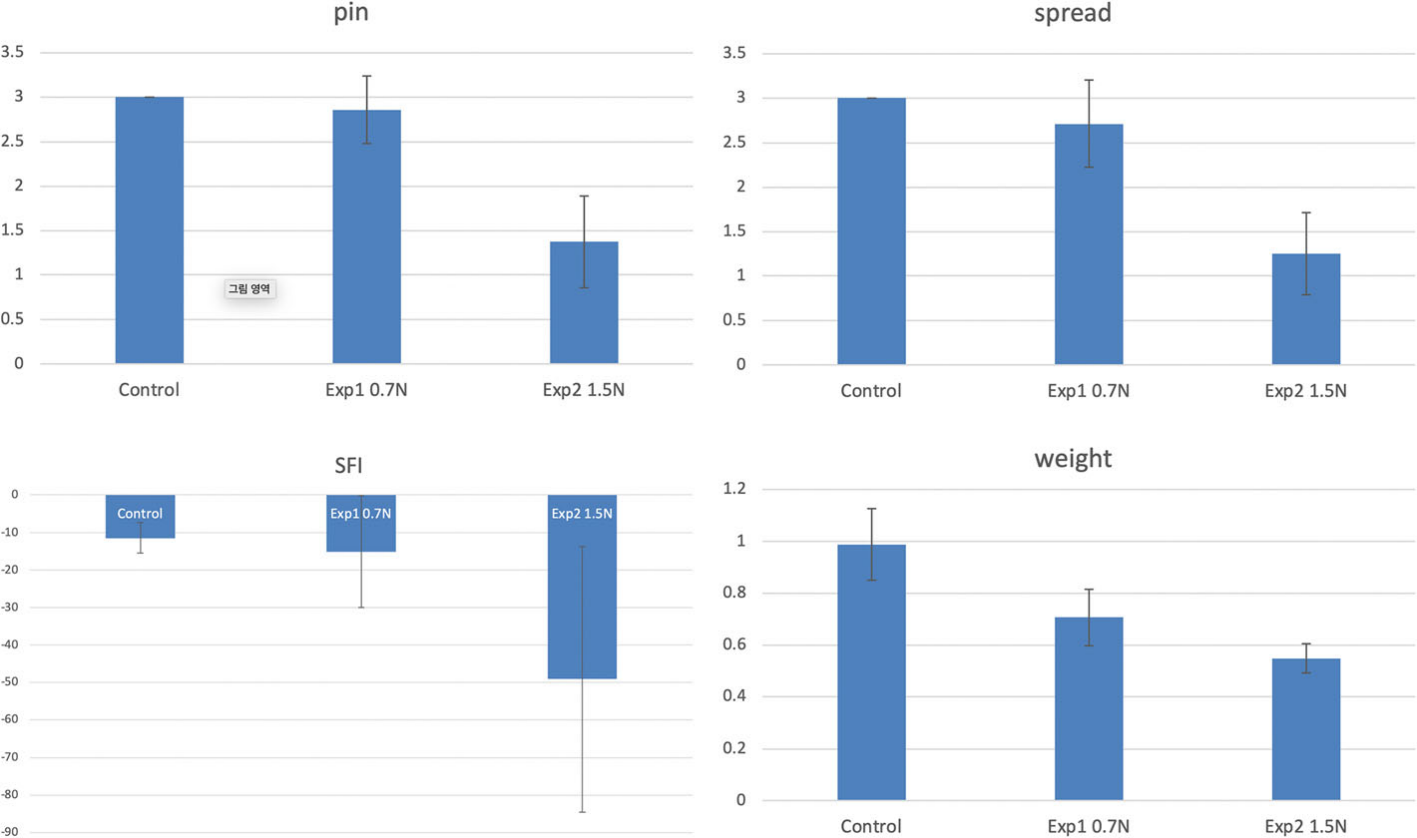
Results of functional test and muscle weight comparison. (**a** pinprick test, **b** toe spread test, **c** sciatic function index from walking track analysis, **d** gastrocnemius muscle weight comparison)

Footprints were obtained before testing pinprick and toe-spreading test (Fig. 3). The average value of pinprick test was 3.0±0, 2.86±0.37, and 1.38±0.51 for groups A, B, and C, respectively (Fig. 4a). Mann-Whitney test showed significant differences between groups A and C (*p*<0.001), and between groups B and C (*p*=0.001). There was no statistical difference between groups A and B (*p*=0.467). The average values of the toe-spreading test were 3.0±0, 2.71±0.48, and 1.25±0.46 for groups A, B, and C, respectively (Fig. 4b). Mann-Whitney test showed significant differences between groups A and C (*p*<0.0001), and groups B and C (*p*=0.02). There was no statistically significant difference between groups A and B (*p*=0.20). The SFI values were −11.48±4.0, −15.09±14.83, and −49.11±35.41 for groups A, B, and C, respectively (Fig. 4c). Mann-Whitney test showed a significant difference between groups A and C (*p=0.010*). However, there were no statistical differences between groups A and B, nor B and C (*p*=0.955 and 0.040, respectively). Muscle weight ratio values were 0.98±0.13, 0.70±0.10, and 0.54±0.056 for groups A, B, and C, respectively (Fig. 4d). The results of the Mann-Whitney test showed significant differences between groups A and B, and also groups A and C. However, there was no statistical difference between groups B and C (*p*=0.021>0.0167) (Table 1).

**Table 1:** Total number of axons, and axons/mm2 in groups A (sham), B (0.7 N tension stretch), and C (1.5 N tension stretch)

Group		Total axon	Axons/mm^**2**^
**A**		3622.33±492.56	11379.89±1547.45
**B**		1316.00±69.16	4134.33±217.29
**C**		712.17±69.49	2237.33±218.31
*p*-value		0.001	<0.001
*p*-value between the groups	A and B	0.004	0.004
	A and C	0.002	0.002
	B and C	0.004	0.004

### Histomorphometric evaluation

**Fig. 5:**
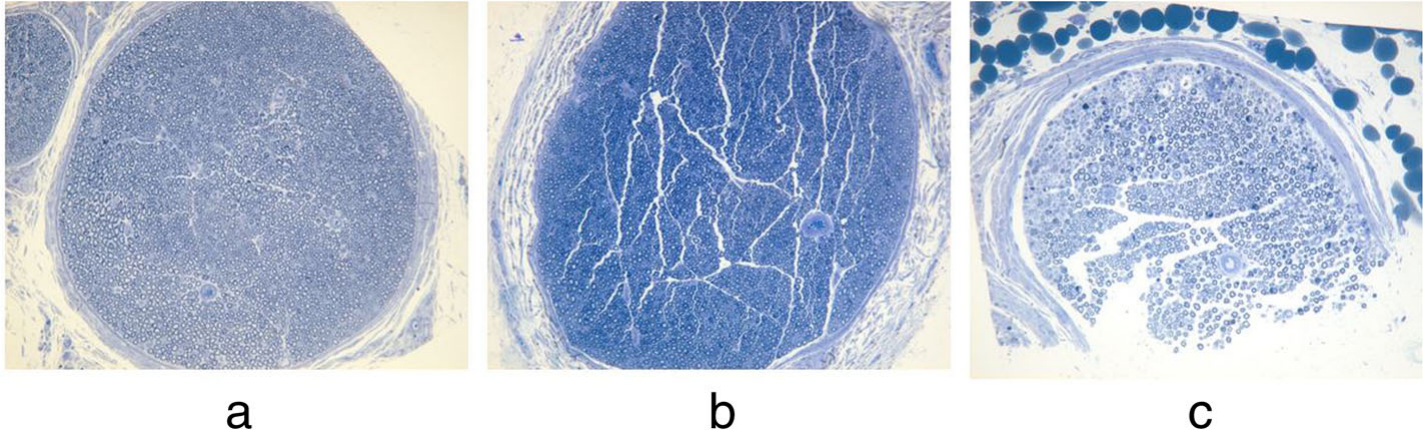
Cross-sectional photographs showing differential axonal damage of each experimental group with toluidine blue staining of ×100 magnification. (**a** intact axons and perineurium of group A, **b** partial disintegration of axons and perineurium of group B, **c** moderate damage of axons with disintegration with swollen myelin of group C)

**Fig. 6:**
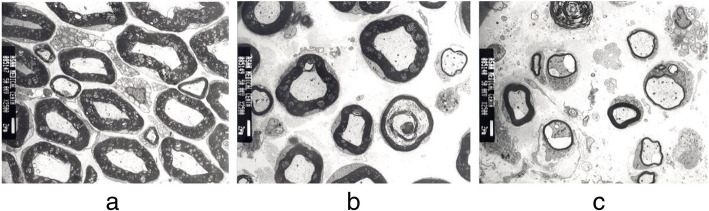
Transmission electron microscopic photographs showing axonal changes of each group with ×2500 magnification. (**a** intact axons and myelins of group A, **b** atrophic axons and myelins with inter-axonal space of group B, **c** disintegrated axons with severe atrophic myelins of group C)

The low- and high-grade tensile forces caused 20% and 40% longitudinal elongation of the nerve fibers, respectively. The longitudinal sections showed reduced fiber width in groups B and C in comparison to group A. Toluidine blue staining prior to TEM examination showed the degree of axonal damage according to the strength of the tension (Fig. 5). Moreover, the degree of the separation between the fibers was increased in partial area of group C comparing to the other two groups. The average numbers of axons and axonal density with statistical evaluations are described in Table 1. TEM examination showed marked disintegration of the axons and surrounding tissues in group C. The spaces between myelinated axon were sparser in groups B and C than group A (Fig. 6). The mean myelin thickness, diameter of the myelinated and unmyelinated axons, and G-ratio are shown in Table 2. There was a significant difference between groups A vs B (*p<0.05*) and A vs C (*p<0.05*) in diameter of myelinated and unmyelinated axons and G-ratio and between group A vs C in mean myelin thickness (*p<0.05*). However, there was no statistical difference in other groups.

**Table 2:** Myelinated axon thickness, unmyelinated axon thickness, G-ratio, and myelin thickness in groups A (sham), B (0.7 N tension stretch), and C (1.5 N tension stretch)

	Group	Mean
**Myelinated axon thickness (μm)**	**A**	5.82±0.30
	**B**	6.23±0.23
	**C**	3.97±0.34
**Unmyelinated axon thickness (μm)**	**A**	3.95±0.22
	**B**	4.50±0.17
	**C**	2.98±0.25
**G-ratio**	**A**	0.68±0.014
	**B**	0.72±0.011
	**C**	0.75±0.016
**Myelin thickness (μm)**	**A**	1.85±0.11
	**B**	1.72±0.08
	**C**	0.98.12

## Discussion

In this study, neurapraxia or axonotmesis by Seddon [16] and Sunderland [17] classification was simulated by traction injury with spring-balance device. Nerve injuries by traction or compression result in ischemia, intrafascicular edema, or demyelination. Usually, nerve damage showed slower recovery than other tissues and may take weeks to months for recovery [18]. In 2008, Mazzer et al. introduced a nerve injury device capable of applying constant and uniform loads in different quantities and varying lengths which is an important factor in inducing reproducible injuries [19]. However, traction injury of sciatic nerve was not simulated by Mazzer’s device. In this experiment, we could develop traction injury model of the sciatic nerve with various traction power, which might be utilized in the clinical situations.

Analyzing the sensory reaction of the rats was very challenging since they struggled during the test. To make it easy to examine the pinprick and toe-spreading tests, we waited 5 min to calm the animals down after gait analysis. The pinprick results showed no statistical difference between groups A and B. One of the possible explanations might be the traction power of 0.7N was reversible without sensory damage. Traction power over 1.0N could damage the sensory nerve irreversibly and impair function for long time. Because this study was terminated in 2 weeks after traction injury, long-term observation study is required to vindicate the reversal capacity of the traction damaged nerve.

In the walking track analysis, it is crucial to let the rats walk along the walking pathway freely before recording the steps. When the animal is placed in the pathway, they often tend to stop while pressing the entire footpad and heel-down, creating a false, untypical long print length. Also before entering the darkened pathway, the rat may stand up, putting all its weight onto its hind limbs and create a false long footprint [20]. A corridor was tailor-made to minimize the known errors, which was composed of a walking pathway, start-floor area, and end-floor area. The floors prevented false prints, and the rats were exposed to the printing paper only while walking down the pathway. Moreover, obtaining clear footprints might be challenging due to contractures, autotomy, smearing of the print, and dragging of the tail [14, 21]. The SFI was quite poor in group C which meant severe damage of the motor nerve.

In the previous study, partial weight bearing was started during the second week and the spreading of the toes during the third week post-injury [22]. Therefore, recognition and precisely marking the key points were reported to be very challenging in the first 2 weeks. Hence, in the present study, the test was performed 2 weeks post-injury. The results showed no significant difference between groups A and B, and groups B and C, which may be explained by the short recovery period after the nerve injury.

Kobayashi et al. reported that the gastrocnemius and extensor digitorum longus muscles were suitable to evaluate denervation effect of the experimental animals 6 months after damage [23]. However, denervation leads to adipose and fibrous tissue formation which increases the total mass, so it is hard to dissect the muscle only [24]. Therefore, in this study, samples were collected within the minimal healing period after the injury which minimizes fibrosis and adipose tissue formation. Moreover, the muscles were excised very carefully, eliminating any other tissues from the samples in an effort to improve the reliability of the results, which showed significant difference among the three groups.

Mazzer et al. showed that large diameter fibers were damaged first and more intensely following an injury, and small diameter fibers were relatively preserved [19]. However, after a certain threshold, the small fibers are also broken, and the damage increases rapidly in proportion to the load applied. The lower range of stretch which produces histological changes has been reported to be between 4 and 50% of the initial length [25, 26]. In our previous pilot experiment for this study, rupture of the sciatic nerve started at 3~6 N tensile strength from which point the forces went directly inside the nerve and causing complete disruption. The difference of the myelin thickness showed that fibers undergoing axon atrophy predominated over fibers undergoing demyelinization, thus indicating that the predominant type of injury produced by tension is axonotmesis. The differences of the myelin thickness and G-ratio in the three groups showed that more tensile strength results to more severe damage, which as a result, it changed the myelin thickness and G-ratio. Moreover, separation gaps were observed in the experimental groups which can be explained by the disruption of the vessels and discontinuity of nerve fiber due to the tension injury. Also, disruption on the vessels will cause hematoma, and as a result, narrow partial areas and separation gaps were observed in the longitudinal sections. Nonetheless, same pattern of damage was observed to some extent in the control group, which may be attributed to nerve handling, tissue harvesting, and histological preparation artifacts. Irregular round-shaped axons with irregular thicknesses of myelin were observed in sciatic nerve samples which made it difficult to estimate the diameter of axons and the total number of the axons. In this study, four different cross sections of the samples were used to measure the total number of axons and axon density. Some investigators applied the three-window sampling method to measure the total number of axons [27]. In this technique, all fascicles that could contain three separate rectangular windows of 0.012 mm^2^ were included. One of these three windows was placed in the center, whereas the other two were randomly positioned on both sides at the periphery of the fascicle in the opposite direction. In the present study, both techniques were used and compared. The results showed no significant difference between the two methods. Nonetheless, Cai et al. reported significant differences between the data obtained by the three-window sampling methods and the conventional method [28]. In this study, the tension injury decreased the total number and the density of axons, which was negatively correlated with the amount of the tensile strength applied.

## Conclusion

The present study demonstrated that the nerve traction injury in the rat sciatic nerve damaged the motor and sensory function and axonal integrity. The amount of functional nerve damage was proportional to the amount of traction power and dependent on the initial tensile strengths (0.7N and 1.5N) which caused 20% and 40% elongation of the sciatic nerve, respectively. The functional recovery was approximately 85% vs 50% in the 0.N and 1.5 traction injury, respectively.

## Data Availability

All the experimental data were retrieved from animal experimental center of Asan Medical Center.

## Abbreviations

SFI: Sciatic function index
(E or N) PL: (Experimental, normal) print length
TS: Toe-spreading
IT: Intermediary toe-spreading
TEM: Transmission electron microscope
